# The Aviation Paradox: Why We Can ‘Know’ Jetliners But Not Reactors

**DOI:** 10.1007/s11024-017-9322-4

**Published:** 2017-06-07

**Authors:** John Downer

**Affiliations:** School of Sociology, Politics and International Studies (SPAIS), University of Bristol, 11 Priory Road, Bristol, BS8 1TU UK

**Keywords:** Engineering, Reliability, Risk, Safety, Regulation, Technology assessment, Nuclear energy, Civil aviation, Jetliners, Reactors

## Abstract

Publics and policymakers increasingly have to contend with the risks of complex, safety-critical technologies, such as airframes and reactors. As such, ‘technological risk’ has become an important object of modern governance, with state regulators as core agents, and ‘reliability assessment’ as the most essential metric. The Science and Technology Studies (STS) literature casts doubt on whether or not we should place our faith in these assessments because predictively calculating the ultra-high reliability required of such systems poses seemingly insurmountable epistemological problems. This paper argues that these misgivings are warranted in the nuclear sphere, despite evidence from the aviation sphere suggesting that such calculations can be accurate. It explains why regulatory calculations that predict the reliability of new airframes cannot work in principle, and then it explains why those calculations work in practice. It then builds on this explanation to argue that the means by which engineers manage reliability in aviation is highly domain-specific, and to suggest how a more nuanced understanding of jetliners could inform debates about nuclear energy.

## Introduction

### Reliability and Governance

In a recent expert forum on nuclear energy and climate change, one participant—a manager involved in Chinese infrastructure—compared the engineering challenges of nuclear energy to those of civil aviation. Having acknowledged the existence of public concerns about reactors, he confidently asserted that the challenges of nuclear energy were amenable to the kinds of engineering and management practices that led to the development of safe air travel, and predicted that public confidence would come to acknowledge this in time. We can build safe jetliners, in other words, so we can build safe reactors.

It is not uncommon in policy discussions about technological safety to see the aviation and nuclear spheres invoked in ways that suggest they are equivalent or analogous in respect to their governmentality (e.g., Bier et al. 2003), and it is easy to see why. The safety challenges of reactors parallel those of jetliners in many ways. Both are similarly complex systems that demand similarly extraordinary levels of reliability;^1^ the industrialized world has been manufacturing both for roughly the same amount of time; and most polities manage their safety through ostensibly similar institutions and practices.^2^ In the US, for example, reactors and jetliners are each the responsibility of a dedicated government regulator (the Nuclear Regulatory Commission [NRC], and Federal Aviation Administration [FAA], respectively) that performs various safety-related functions; the most fundamental of which being the formal safety assessment of new designs (airplanes and reactors) prior to their use.

Jetliners and reactors are important because they represent the vanguard of a burgeoning number of complex systems with the potential to fail catastrophically enough that their performance warrants public scrutiny. The emergence of these systems (henceforth referred to as “critical technologies”) has transformed technological safety into an important yet under-recognized metric of modern governance. Experts make strong claims about the future performance of reactors^3^ and jetliners. (With all critical technologies, it is necessary to make predictive reliability assessments. It would not be acceptable to design either jetliners or reactors with the understanding that their reliability will only become apparent over time.) And policymakers invoke these claims in deliberating significant policy questions pertaining to transport and energy.

Experts are able to make strong claims about technological performance because the safety of critical systems is understood as a function of their ‘reliability’ (i.e., we prove that such systems are safe by proving that they will not fail catastrophically)^4^ and, at least on an institutional level, reliability is understood to be an objectively ‘knowable’ property of artifacts. Policymakers are encouraged, and often obliged, to treat expert reliability assessments as established facts: the product of an objective process with universal principles that is applicable across technological domains (see, e.g., Hilgartner 2007: 154).^5^

Perhaps the clearest manifestation of this is in debates around nuclear energy, the political viability of which has long been premised on declarative, formal assurances that meltdowns—much like meteor-strikes and alien invasions—are too improbable to merit genuine policy consideration (Rip 1986: 7–9; Fuller 1976: 149–186; Ramana 2011). This conviction, which is entrenched enough to have weathered several seemingly-disconfirmatory meltdowns,^6^ is a crucial component of every nuclear cost-benefit calculation, contingency plan and environmental analysis. It is fair to say that Western societies would make different technological choices if they were less than entirely confident in their reliability assessments (see Downer 2016).

For all the significance afforded to expert safety assessments of critical technologies, however, there are compelling reasons to imagine they do not merit the confidence invested in them. Nuclear energy’s troubled history, for instance, conspicuously is at odds with the idea that reactor performance is definitively predictable. And while it is undoubtedly true that many expert engineering calculations justifiably warrant great authority—bridge-building societies would be in trouble if they doubted expert calculations of steel’s tensile strength—reliability is an unusual variable, in that it is the expression of an absence (i.e., of failure): a property that makes it problematic to measure in the manner required by safety-critical technologies (i.e., predictively, at extremely high levels).

In what follows, this paper will outline the argument that reliability calculations of critical technologies are epistemologically implausible, and then explore it in relation to reactors and jetliners. Reactors are important in the context of this argument because reliability calculations are almost uniquely consequential in a nuclear context, due to the potentially extreme consequences of meltdowns (see Downer 2016), yet the validity of those calculations cannot be tested empirically, even long after reactors enter service. (Reactors are too few in number and too varied in design to ever accrue enough service data to provide a statistically significant test of the reliability predicted of them.) Jetliners are important in this context for a very different reason. This is that, almost uniquely among critical technologies, their reliability (and thus the validity of expert calculations that anticipate that reliability) *can* be assessed empirically after they enter service (because we build large numbers of near-identical jetliners), and, confoundingly, they appear to be as reliable as calculations predict.

The reliability of jetliners speaks directly to the capabilities of technology regulators, and has wide ramifications for nuclear governance. For if aviation regulators have mastered the art of ultra-high reliability prediction, then it seems intuitive to infer that their counterparts in other spheres might have achieved the same. If we can build safe jetliners, then why not safe reactors?

### Outline

Drawing on ideas from the Science and Technology Studies (STS) literature,^7^ this paper will propose an unorthodox account of critical-technology engineering and its assessment: an account that speaks to the authority of expert safety-claims and the limits of engineering rationality.

The following section, (part 2, below), outlines the ostensible logic of critical-technology reliability prediction, and explains why this logic should be suspect. It argues that such predictions are derived from tests and models that are limited, epistemologically in ways that preclude the kind of certainty that regulators require. It further explains that critical technologies ought to pose an unrealizable design challenge for the same reason that they pose an impossible assessment challenge. Having outlined a principled argument for why ultra-high levels of reliability ought to be impossible to achieve and to assess, it concludes by noting that jetliners demonstrably achieve such levels, and that aviation regulators demonstrably succeed in predicting them.

The subsequent section, (part 3), explores the tension between the dubious epistemology of reliability calculations and the manifest reliability of jetliners. The key to resolving this tension, it contends, lies in understanding that jetliners are neither assessed nor designed in the manner that engineers and regulators purport. To this end it introduces two institutional norms (‘recursive practice’ and ‘design stability’), which, it argues, allow the civil aviation sphere to manage ultra-high levels of reliability by leveraging service experience rather than tests and models.

The final section, (part 4), argues that civil aviation’s achievement is not generalizable to other technological spheres. By examining the practices outlined in the previous section in relation to Concorde (briefly) and the nuclear industry (in more depth), it argues that the ability to build and assess reliable jetliners is highly contingent and does not imply a similar competency with regard to reactors. The nuclear sphere, it concludes, is actually governed through the idealized and epistemologically-implausible processes by which aviation only purports to be governed, and this should be recognized in public discourses around reactor safety.

## Epistemological Limits

### Assessment in Principle

Assessing the performance of either a new reactor or jetliner is a forbiddingly complex enterprise governed by a kaleidoscope of esoteric documentation and procedure. In both cases it takes hundreds of highly qualified people several years to complete, costs millions of dollars, and generates literally tons of paperwork. The various practices, interpretations and conventions involved in this process offer rich material for sociological scrutiny (see, e.g., Downer 2011a; Perrin 2005). For the purposes of this paper, however, I will focus not on the nature of the task but on its epistemology.

The epistemology of technology assessment is interesting because, as noted above, reliability is an unusual property of artifacts. Unlike mass, density, volume, or almost any other variable, it is a ‘negative’ property: being defined by the absence of failure. It is also an unusually contextual and interpretive property: being a function of the circumstances in which one expects a system to function and the point at which one deems it to have ‘failed.’ These properties can create complex issues for experts who would quantify the reliability of any system, but their significance is greatly accentuated when the reliability required is of the levels expected of critical technologies (let us call it ‘ultra-high’).

There are several reasons why reliability calculations pose unique epistemological challenges in the context of critical technologies. Perhaps the most fundamental is that, as noted above, such calculations have to be *predictive*. In most circumstances reliability is an actuarial property: one that engineers extrapolate from service data. Reliability assessments of infantry rifles, for example, are simply expressions of how often those rifles have failed in the past, combined with some basic *ceteris paribus* assumptions about the future (that the circumstances of their use and manufacture will remain constant, for instance).^8^ Critical technologies cannot be assessed in this way. Their safety performance needs to be assessed before they are built; usually as a condition of them being built. In these circumstances, experts have to make predictive, forwards-looking calculations that are not grounded in past service data.

The fundamental basis for these predictive calculations are bench tests performed in advance of actual service. This poses a problem, however, because critical systems demand reliabilities that are higher than tests can claim to demonstrate. A common requirement for critical sub-systems, for example, is an established mean-time-to-failure of not less than a billion hours. But a test system would have to run, failure-free, for over 114,000 years to demonstrate this level of performance (Rushby 1993).^9^ Running many tests in parallel reduces this number but not to a practicable level. Instead, therefore, critical systems are designed in ways that make them measurably more reliable than their constitutive sub-systems.

To this end, engineers invoke a variety of design stratagems, the most fundamental of which is redundancy: the use of extra elements integrated in ways that allow one to fail without imperiling the wider system. A simple example would be the fact that jetliners have more engines than they require for flight, so that if one should fail there will still be enough power to effect a safe landing.^10^ Redundancy is a useful design technique for reliability, but it is also an invaluable assessment tool. This is because it allows regulators to combine empirical tests with theoretical models to demonstrate much higher levels of reliability than would be possible via testing alone (Downer 2009, 2011a).

The principle behind this is simple. When two identical and independent elements operate in parallel to form a single system, the probability of that system failing can be expressed as the probability that both of its elements will fail at the same time, (i.e., as the two probabilities multiplied by each other).^11^ Redundant elements in a system need only demonstrate a fraction of the reliability required of the system itself, therefore, and this means that tests of those elements can serve as an empirical basis for reliability assertions that are much higher than tests could establish directly.

In broad epistemological strokes, therefore, this is how critical technology assessment works in principle. Regulators test the elements of a system in a lab and then combine the results of those tests, via redundancy calculations, to demonstrate that the system achieves the ultra-high levels of reliability it requires. All formal reliability assessments of critical systems, when stripped to their logical core, are ostensibly invoking this same basic combination.

In practice, however, the process is much more complicated than it appears.

### The Problem of Relevance

The devil, as always, is in the details. The logic outlined above hinges on tests and models, but academic observers of engineering practice—most notably those working in the STS tradition (e.g., Wynne 1988; Collins and Pinch 1998; MacKenzie 1990)—have routinely found that tests and models struggle to capture the ambiguities of real performance. There are various arguments offered for why this is so, but the most fundamental relates to what philosophers sometimes refer to as ‘the problem of relevance.’

The problem of relevance speaks to the difficulty of knowing how closely a representation of a phenomenon, like a test, maps onto the real phenomenon that it purports to represent. To know that a test is providing accurate information, for example, testers must determine whether it sufficiently mimics the real world in all the ways that ‘matter’; yet there are a potentially infinite number of such determinations and no rigorous way of assessing their accuracy or completeness. So it is that close studies of engineering tests frequently find that meaningful doubts and uncertainties surround those tests’ real-world implications (e.g., Pinch 1993; MacKenzie 1996; Downer 2007). MacKenzie (1990), for example, shows how engineers directly involved in testing US ballistic missiles were privately skeptical about extrapolating from tests to operational performance due to their awareness of the tests’ hidden contingencies and assumptions.

Studies of engineering models (e.g., MacKenzie 2001) illustrate the same shortcomings for the same underlying reasons. The straightforward mathematics of redundancy outlined above, for instance, become much more ambiguous if we consider that redundant elements might fail for interconnected reasons, or that a failure in one element might ‘propagate’ to others (Downer 2011a; Sagan 2004). Such relationships are impossible to idealize perfectly, and cannot be tested empirically without shackling the models with the relevance questions that plague tests themselves, and with the same limitations (i.e., on the levels of reliability they can plausibly demonstrate) that the models are used to transcend.

It follows from the above, STS scholars argue, that even the most rigorous engineering tests and models necessarily contain uncertainties, but critics sometimes dismiss this point as dogmatic and inconsequential in relation to engineering. Vincenti (1990), for instance, describes engineering as a practical discipline that is more interested in ‘utility’ than in ‘truth,’ and argues that its ambitions are rarely undone by the esoteric dilemmas that exercise STS scholars. Constant (1999) makes much the same point. For all the misgivings of epistemologists, he observes, there is no denying that “most of our stuff works most of the time.” There is undeniable merit to this position. The philosophical niceties of representation have not kept engineers from realizing pocket supercomputers, space rockets that land themselves on floating barges, and much else besides.

At the same time, however, there are compelling reasons to believe that the problem of relevance matters to reliability calculations of critical technologies in a way that it doesn’t to other engineering endeavors. This, in essence, is because unlike most engineering endeavors, interrogating ultra-high levels of reliability is more akin to a search for ‘truth’ than a search for ‘utility.’ In many ways reliability can be understood as an expression of ‘certainty’ (i.e., that failures will not occur). So ultra-high levels of reliability imply commensurately high levels of certainty: it makes little sense for nuclear experts to say they are ‘modestly’ sure that the chance of a meltdown is ‘extraordinarily unlikely.’ When engineers are trying to establish that a system will operate for billions hours without failure, therefore, then even very marginal ambiguities become meaningful. For these calculations to be accurate, the tests and models on which they are based need to be almost perfectly comprehensive and faultless: something that the problem of relevance precludes.

It follows from this that the reliability of jetliners and reactors should be unknowable. Given that the challenges of assessing the reliability of a critical technology are closely aligned to the challenges of designing one to be ultra-reliable, moreover—the key to both lying in identifying potential weaknesses—then there are good reasons to imagine that new reactors and jetliners should be unreliable for the same reason that their reliability is unknowable. So it is that investigations of critical-technology accidents frequently identify failure conditions that engineers missed because their tests and models imperfectly represented the real world. Fukushima’s safety assessments, for example, were found to have hinged on erroneous assumptions about tsunami risks and misplaced premises about the independence of redundant backup-generators, and much else besides. (Elsewhere I have referred to failures that arise from erroneous engineering beliefs hidden in the logic of tests and calculations as ‘epistemic accidents’ (Downer 2011b)).

This is all to say that, from an epistemological perspective at least, it is almost axiomatic to conclude that knowledge-claims and practices requiring extraordinary degrees of fidelity from tests and models ought to be unrealizable. The task of predicting the performance of a complex system, with many variables and contingencies, over a long timeframe, to an exacting degree of accuracy, simply requires too many judgments to be made with too much perfection to be plausible. There is no way that experts should be able to deduce from tests and models that a yet-unrealized jetliner or reactor will be reliable to the extraordinary levels that they claim.

It is a conundrum, therefore, that civil aviation experts appear to do exactly that.

### The Aviation Paradox

The US civil aviation sphere exemplifies the practices and assumptions of modern critical-technology assessment. A state regulator, the FAA, has to approve (“type-certify”) each new jetliner design as safe (interpreted as ‘reliable’) before that design is allowed to carry passengers.^12^ To this end it performs an exhaustive series of analyses wherein it assesses each system’s reliability via a process that treats reliability as an objectively measurable variable that can be definitively established in advance of service data. The levels of reliability the FAA requires are extremely demanding, meanwhile, with each ‘safety-critical’ system needing to demonstrate a mean-time-to-failure of over a billion flight-hours.^13^ And the formal justifications of how this figure established conform neatly to the ‘test and multiply’ template outlined above (i.e., regulators invoke systems-level models to combine the results of bench tests performed on individual elements) (see, e.g., NTSB 2006).

In keeping with the argument above, moreover, any close inspection of the FAA’s assessment calculations reveals that they are rife with problematic relevance judgments. Take, for example, the FAA’s ‘birdstrike’ tests, wherein it interrogates an engine’s ability to ingest errant avians by the (seemingly) simple expedient of spooling the engine up to full power and firing birds into it from a cannon. Regulators go to elaborate lengths to make these tests as ‘relevant’ as possible: using freshly killed birds, for instance, because thawed birds lose moisture. For all their efforts, however, long-running debates still surround many aspects of the tests. The types of birds used are contested on relevance grounds, for example, as are their numbers; the velocities at which they are launched; and much else besides (see Downer 2007). The FAA’s redundancy calculations are no less problematic. It may sound straightforward to add a redundant engine to the wing of a jetliner, for example, but this simplicity dissolves if we consider the fact that multiple engines can fail for common reasons,^14^ or explode in ways that jeopardize the whole airplane (see Downer 2011a).^15^

It follows from this that the FAA’s type-certification assessments ought to be groundless. Logically speaking, experts cannot make confident ‘one-in-a-billion’ assertions based on a vast pyramid of tests and models that are contested and uncertain at every level. Yet it is difficult to deny that the assessments have usually been accurate. We can say this with some confidence because when the FAA has approved airplane types in the past, manufacturers have subsequently sold thousands of those aircraft to carriers, which have then operated them almost continuously for years.^16^ Over time, therefore, many airplane types have accrued so much service experience that even the ultra-high levels of reliability claimed of them can be tested ‘empirically’ rather than ‘predictively’ (i.e., their reliability can be inferred statistically from the service data).^17^ And for most modern civil aircraft—with a telling exception that I will discuss below—the service data have confirmed the regulator’s assessments. Jetliners still crash, it is true, but extraordinarily infrequently relative to the number of operational hours they accrue, and rarely because of reliability issues.^18^ Their performance is easily congruent with the levels implied by reliability calculations. There is compelling evidence, in other words, that civil aviation experts are achieving near perfect results on the basis of knowably imperfect tests and models.

To understand how this is possible, it is necessary to understand that the rhetoric of aviation assessments is misleading, as experts in this sphere do not manage reliability in the manner they purport.

## Reliability in Practice

### Design Stability

Aviation experts are able to negotiate the indeterminacies of tests and models for a surprisingly straightforward reason: they do not approach reliability through tests and models. The FAA, in its rhetoric and documentation, presents its assessments as being derived from examinations of the systems themselves; as if the reliability of a system is an inherent property of the design that tests and models can extract. In practice, however, it is truer to say that the assessments are not examining the system itself so much as the service history of its predecessors. Put very simply, the FAA examines how earlier systems have performed in the past; satisfies itself that proposed changes to those systems will not negatively affect that performance; and then assumes that the new systems will be about as reliable as their antecedents. The process is actuarial more than it is predictive.

Regulators are able to assess new jetliners in this way because new jetliners are only ‘new’ in an extremely limited sense. Large civil aircraft undeniably change between ‘types,’ but they change so incrementally that it is highly misleading to think of a new jetliner as an untested technology with no meaningful service record. The civil aviation establishment is intensely suspicious of change. Modern jetliners look almost identical to untrained observers for reasons that go far beyond the pressures of technological determinism.^19^ They look alike because new airframes, and the elements that comprise them, are derivative their processors to a degree that would be extraordinary in almost any other engineering context. Airplane manufacturers routinely market their designs as revolutionary, but relative to their peers in other technological spheres they are the Tories of the engineering world: they believe in progress, but only by consecrating traditions and building on the hard-earned wisdom of their predecessors.

Since the word ‘conservatism’ is already used in engineering circles to indicate generous error-margins, I will refer to this industry-wide norm as ‘design stability.’ It can be understood as a deep institutionalized reluctance to embrace new designs, architectural principles or materials until they have been exhaustively explored in other contexts (in the aviation sphere this is invariably the military). The introduction of ‘fly-by-wire’ controls and new composite materials to jetliners—two of the most significant civil aviation innovations of recent decades—illustrate the strength of this norm. Aviation is a competitive market, and both technologies promised very significant commercial advantages to operators (by shedding weight, and thus fuel costs), yet manufacturers waited until both had been used for decades in military aircraft before introducing them into commercial airframes.^20^ Despite the fact that manufacturers marketed both as radical innovations, neither could really be described as untried technologies that were only knowable through tests and models.^21^

There is a meaningful sense, therefore, in which manufacturers have been carefully refining the same basic airframe ‘paradigm’ for almost 70 years. And, because there is statistically significant evidence of how airframes built on this paradigm have performed in service, regulators can leverage this stability to make useful predictions about new airframes.^22^ Aviation experts can avoid basing their assessments on tests and models, in other words, because they have service data on which to draw instead. Epistemologically speaking, the reliability of jetliners is established in much the same way as that of rifles.

### Recursive Practice

The process of achieving (as opposed to assessing) reliable designs is worth considering separately. As noted above, the inherent ambiguities of tests and models are liable to cause accidents as well as assessment errors, and, as such, pose a dilemma for an airframe’s designers as well as for its regulators. While regulators can make useful reliability predictions on the basis of good service data and a strong commitment to design stability, however, ‘achieving’ ultra-high levels of reliability requires something more: a commitment to learning and incremental refinement.

The aviation industry owes much of its current level of safety to decades of assiduously mining its misadventures. It explores its failures for insights that allow it to hone its understandings, and thus its designs. A litany of aviation accidents and near misses have provided insights that have led to subtle, and often highly specific, refinements of airframe designs (and of the tests and calculations that inform those designs) (see Downer 2011b).^23^ The reason that all airplanes have oval windows, for example, dates back to the loss of two De Havilland Comets in 1953 and 1954. The accidents revealed that the airplane’s rectangular windows were inducing stress fractures at the corners: something that pressurization tests had failed to reveal because they imperfectly represented some significant variables (Faith 1996).

Constant (1999) calls this incremental learning ‘recursive practice,’ and like design stability, it is deeply ingrained in the culture of civil aviation.^24^ The industry, often with state support, goes to extraordinary lengths to identify the causes of its failures, sometimes spending years reconnoitering inhospitable ocean floors in pursuit of a crucial design insight. Few, if any, engineering endeavors can compare.

### The Foundations of Safety

We might say that ‘service history,’ ‘design stability,’ and ‘recursive practice’ together make a three-pronged scaffold on which the epistemology of civil aviation rests. Alone they do not count for much, but in combination they allow manufacturers to defy the epistemic limitations of tests and models to achieve, and predict, extraordinary levels of reliability. A deep well of service experience creates a set of occurrences (some catastrophic) that tests and models alone could never predict. Investigating those occurrences reveals insights that allow for design refinements. And keeping designs stable (in other respects) helps ensure that those refinements continue to be relevant; thereby making the effects of refinement cumulative and useful.

**Fig. 1:**
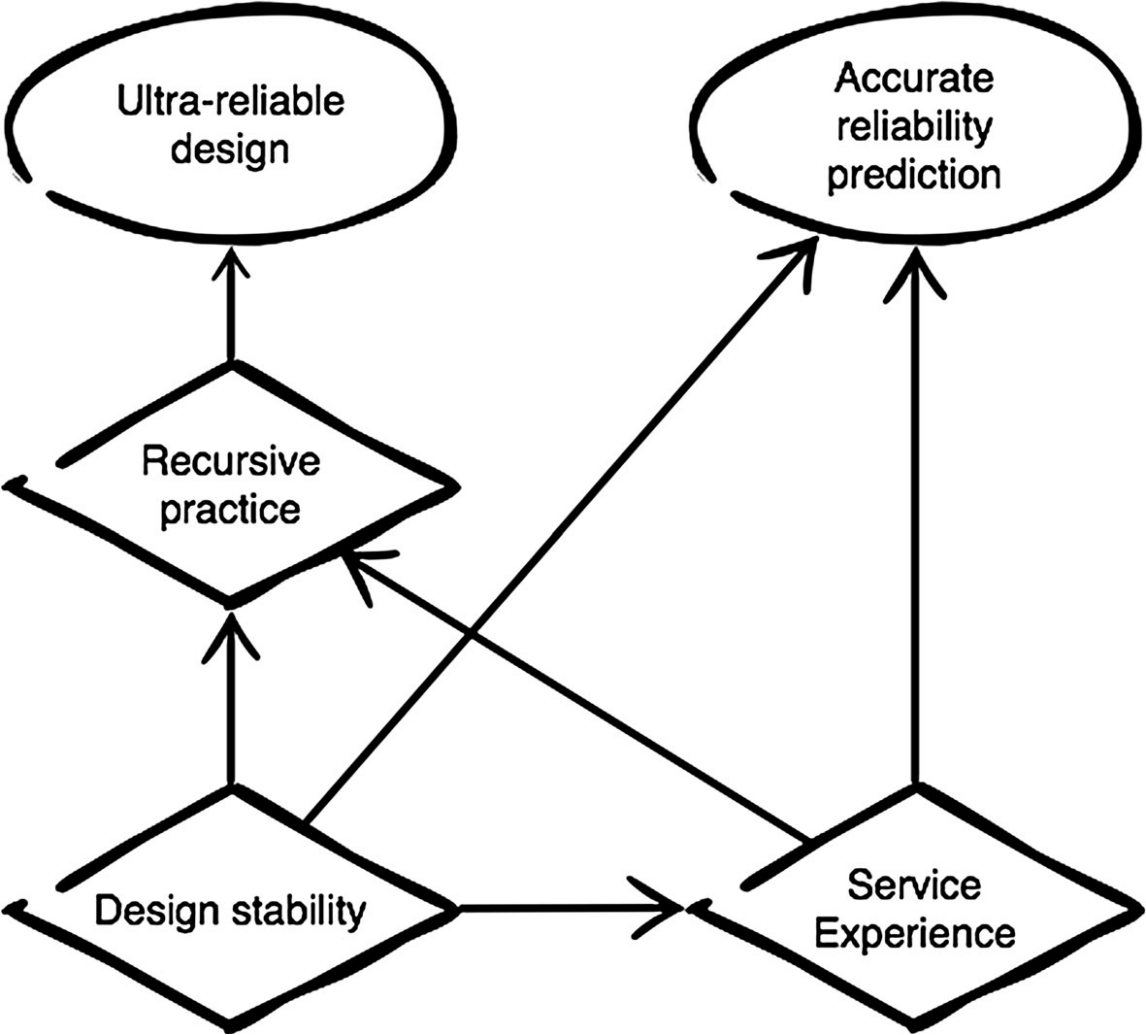
The foundations of safety

Figure 1 maps the relationships between these principles as a crude schematic. It illustrates several points worth reiterating:

1. *Design stability helps create relevant service experience.* The greater the similarity between past and present designs, the more useful and representative the industry’s service history becomes to experts hoping to predict the performance of the future aircraft.
2. *Design stability and extensive service experience are both necessary preconditions for successful recursive practice.* Service experience is necessary for recursive practice because kinds of misconstruals that evade expert tests and models usually only become apparent in rare and unusual circumstances, thus it takes many operational hours to draw them out. Design stability is necessary because the insights that engineers glean from investigating their misconstruals tend to be highly specific rather than broadly generalizable, thus many become irrelevant when designs change. Hoarding insights means keeping systems stable, in other words, even if it means forgoing or delaying innovations that promise commercial benefits.^25^
3. *Recursive practice is a necessary precondition for ‘achieving’ the levels of reliability required of critical systems, but not for ‘predicting’ that reliability.* All that is needed to predict the reliability of a new system is compelling evidence that it is substantially similar to a previous system, combined with good data on how often that previous system has failed in the past. Recursive practice only becomes important insofar as engineers are looking to improve the safety of a system.

## Generalizability

### The Exceptional Jetliner

The picture of aviation design and assessment outlined above implies that the way that policymakers think about critical technology governance is misleading. By this view, aviation regulators did not learn the secret to predicting the reliability of complex technologies in general so much as they slowly and empirically learned the reliability of a highly specific airframe design. Manufacturers, meanwhile, did not discover a formula for designing ultra-reliable systems in general, so much as they slowly and painfully learned to make a specific airframe design ultra-reliable.^26^ Both assiduously mined the industry’s service record to refine their understanding of a specific design paradigm, and then worked hard not to deviate from that paradigm. Crucially, the fruits of this process are not generalizable across technological spheres or even across technologies in the same sphere. Anyone wishing to hone (or assess) a fundamentally different design to similar level of reliability would have to start almost from the beginning.

A good way to illustrate the truth of this is to consider a civil jetliner that conspicuously violated the standard design paradigm: Concorde.^27^

Anyone looking for an airplane that flaunted the principles of design stability and recursive practice could hardly hope for a better example than Concorde. The trans-sonic airplane broke so radically with the standard airframe paradigm that many aviation historians consider it to be an entirely different class of vehicle to a traditional jetliner (e.g., Loftin 1985; Schrader 1989). Its cruising speed of 1,354 mph—over twice that of any jetliner in operation today—was a remarkable engineering feat, but one with extensive design ramifications. Aerodynamically, for example, it necessitated the ‘double delta’ wing shape responsible for Concorde’s instantly recognizable silhouette. The wings, in turn, forced the plane to land at an unusually steep angle, requiring unorthodox landing gear and a nose that moved so pilots could see the runway. The air friction at high speeds, meanwhile, required special heat-resistant alloys and subjected them to unprecedented fatigue loads. It also required an unprecedented cruising altitude, which led to unique pressurization considerations. As a result of these demands, and many others like them, the airplane embodied a spectrum of ideas, technologies and manufacturing techniques that were new to civil aviation (Owen 2001; Orlebar 1997).

Concorde is often remembered as having an impressive safety record despite its architectural audacity, but this is highly misleading. It entered service in 1976 and retired 27 years later in 2003. During this period it had only one serious accident: *Air France Flight 4590*, which crashed on takeoff from Paris in July of 2000. For most jetliners, 27 years of operation with only one disaster would be an extraordinary record: compelling evidence that ultra-high reliability was compatible with radical innovation. This record needs to be understood in context, however. Concorde had an anomalous market share as well as an exceptional design. Only 14 ever entered service, and even at peak service they flew far fewer hours per day than other jetliners. As a result, it accrued far fewer flight hours than other designs. (It made about 50,000 flights total, whereas the Boeing 737, by contrast, made about 232 million flights over the same period.)

Concorde’s extremely modest service history is essential to understanding its safety. Statistically speaking, its one accident made the airplane an order-of-magnitude less reliable than more traditional airframes. Its designers broke with the conventional design paradigm for civil jetliners and the airplane paid the price in reliability. Regulators were wrong about its safety and should have refused to approve it on the grounds that its design was too unprecedented, (and without the enormous Anglo-French political investment in its success they would likely have done so).^28^ It is telling that no manufacturer has seriously attempted anything similar since.^29^

### Nuclear Insecurities

The inability of civil aviation manufacturers and regulators to achieve with Concorde what they achieved with other jetliners underlines the specificity of their reliability accomplishments. This specificity is important to the nuclear sphere because, as noted, reactors are equivalent to jetliners in important respects and are assessed in an ostensibly similar manner (via a formal reliability calculation overseen by a dedicated government regulator).^30^ When considered in light of the discussion above, however, any apparent equivalences between the aviation and nuclear spheres begin to break down.

To understand the nuclear sphere’s relationship to reliability, it is necessary to appreciate its dearth of statistically significant service experience. The number of active commercial reactors in operation around the world is tiny relative to the number of jetliners. In 2011, prior to Fukushima, there were roughly 400 operational reactors, a number that is so small that—even if each reactor had begun its operational life at the dawn of the nuclear age, and there had never been a single nuclear accident—their cumulative error-free operational hours would be insufficient (by an order of magnitude) to statistically demonstrate the levels of reliability its regulator requires.^31^

This lack of service experience is exacerbated, moreover, by the fact that the nuclear manufacturers evince far less of a commitment to design stability than their counterparts in civil aviation. It would be wrong to say that nuclear architects do not adhere to any common paradigms, but even within the small pool of operational reactors the disparity between designs dwarves that of jetliners.

Reactor designs can be envisioned as a many-branched family tree. At the broadest level, they can be divided into ‘types’ based on their fundamental operational principles (Pressurized Water Reactors; Pressurized Heavy Water Reactors; Boiling Water Reactors; Gas cooled Reactors, Molten Salt Reactors, and several others). In turn, these ‘types’ can be divided into ‘generations,’ each representing very considerable design differences (the industry regularly divides Pressurized Water Reactors into three generations, for example). Even reactors of the same type and generation, meanwhile, are ‘bespoke’ in the sense that they are tailored by their manufacturers (which, again, are more numerous and diverse than in aviation) to suit specific local conditions (such as seismic and flood risks): a process that creates significant variations between otherwise ‘identical’ plants.

The degree of variation between reactors means that the performance of one says relatively little about the performance of others. As was evinced, for instance, in the aftermath of the Chernobyl and Fukushima disasters, when nuclear experts repeatedly highlighted the irrelevance of those designs to the safety of other plants around the world (see Downer 2014).^32^ This lack of relevance further undermines the usefulness of the industry’s already thin service data.

Nuclear’s lack of service experience and design stability have complex ramifications that profoundly shape its relationship to reliability:

1. Firstly, they make it impossible to retroactively assess the credibility of reactor safety calculations. With no statistically relevant service experience, there is no empirical evidence to indicate whether even the longest-serving reactors are as safe or reliable as experts calculated.^33^ (And with so many differences between plants, even those reactors that fail can be dismissed as having very limited significance).
2. Secondly, and relatedly, they keep the NRC from assessing reactors in the same way that the FAA assess jetliners. As discussed, the FAA can usefully predict the reliability of a new airframe, despite the limitations of its tests and models, because: (a) it knows the airframe is very similar to other airframes, and (b) it knows, statistically, how reliable those airframes have been in service. The NRC has neither advantage. It must *actually* predict the reliability reactors in the manner that aviation regulators just *purport* to predict reliability of airframes—from the tests and models, with all their manifest epistemological shortcomings.
3. Thirdly, and finally, they make recursive practice far more difficult in the nuclear sphere than in aviation. Nuclear manufacturers do endeavor to learn from their limited service experience (see, e.g., Perrin 2005: xi), and there are, to be sure, generalizable lessons learned from nuclear mishaps. (Post-Fukushima reactors are unlikely to be designed with all their backup generators underground, for example.) Yet these lessons are of a different order to those collected by aviation manufacturers, which often involve insights into very specific design details in highly uncommon situations.^34^ The large disparities between reactor designs coupled with the small number of reactors in operation mean that the lessons of experience are fewer, and their usefulness far lower, than in aviation. As a consequence, nuclear architects, like nuclear regulators, are also more dependent on tests and models than their aviation counterparts. In theory, this should make reactors much more susceptible to disasters arising from undiscovered misjudgments hiding in their designs (‘epistemic accidents’). It follows from this, therefore, that reactors should be less reliable than jetliners for the same reason that their reliability is less knowable.

This is all to say that design and assessment practices are not equivalent across technological domains, even if they are often presented as such. Nuclear and aviation regulation look like very similar undertakings, and reactors and jetliners appear to present analogous engineering challenges, but the means by which regulators ‘know’ the reliabilities of these systems, and the means by which manufacturers have pursued reliability in their designs, are necessarily different. The aviation sphere, with its expansive service experience and longstanding commitment to design stability, is epistemically privileged in a way that the nuclear sphere is not, and it has leveraged this privilege to make accurate predictions and build safe airplanes in a way that its nuclear counterparts cannot replicate.

There are no shortcuts to achieving or assessing reliability in critical technologies. The safety of modern aviation and the accuracy of its reliability assessments are both inseparable from the industry’s long, slow and sometimes painful history. Those who misunderstand this are prone to adopt an idealized and misleading understanding of engineering knowledge: one that implies a generalizable level of technological comprehension and competence that can be dangerous in spheres where it does not belong. This is important. For while it may be true, as an old adage puts it, that mastery is most often achieved by those who don’t know that failure is inevitable, the same cannot be said of prudent policymaking.

### Considering Uncertainty

The question of what would, in fact, constitute prudent policymaking in these circumstances is complex and deserving of fuller consideration than can be afforded here. Broadly speaking, however, it would require us to consider the choices we would make if we understood that: (a) the probability of reactor meltdowns cannot not be objectively known; and (b) the reliability of reactors is likely to be lower than current assessments predict.

While it is impossible to know where exactly such a reconsideration might lead, it is possible to identify some of the broad policies that might need to be rethought. One, for instance, would be the manner in which reactor safety is analyzed and assessed. Early US reactor safety assessments, such as the 1957 ‘WASH-740’ study, explored the potential consequences of major disasters. As noted above, however, the 1975 ‘WASH-1400’ study put an end to such scrutiny by ‘proving’ that major disasters were too improbable to merit consideration. Recognizing the epistemological uncertainties of such proofs suggests a reevaluation of this approach and, potentially, a return to the former practice of scrutinizing the consequences of accidents as well as their likelihood.

By the same logic, it is likely that we would want to reconsider a range of matters pertaining to ‘resilience.’ The institutional understanding that reactor reliability is objectively calculable not only forecloses the study of disasters, it also curtails the planning around them. As it stands, for instance, the US (as with all nuclear states) does little to prepare for serious meltdowns (again on the basis that they are too improbable to merit serious consideration). This is reflected in evacuation procedures, for example, which are framed around small leaks rather than catastrophic failures (Clarke and Perrow 1996). It is similarly reflected in the NRCs fallout models, which, at the time of Fukushima at least, were only designed to predict radioactive dispersion up to a distance of 50 miles (Lochbaum et al. 2014). More fundamentally, it is reflected in reactor siting decisions. As Perrow (1999) points out, it is common practice for operators to ‘cluster’ multiple reactors on single sites. This clustering has economic and political advantages, but as Fukushima amply illustrated, it creates the potential for a failure of one reactor to jeopardize others. It is arguably a practice that would be difficult to justify in a climate that recognized the evitiability of major accidents.

Most fundamentally of all, of course, we may want to reconsider the viability of nuclear energy in general. In the early 1970s, the shift in the way US reactors were assessed—the formal exclusion of accident analyses on the basis of probability—was driven by a widespread belief that the political viability of reactors would be undermined by a public exploration of potential disasters and their consequences (Rip 1986: 7–9; Fuller 1976: 149–186). Whether this belief was correct at the time, or would still hold today, is an open question. And so long as the safety of reactors is understood to be as knowable (and as achievable) as that of jetliners, that question is unlikely to be answered.

## Footnotes

1 Reactor meltdowns are much more consequential than plane crashes but there are many more jetliners than there are reactors so the per-hour reliability required of each is roughly equivalent.

2 We might say that their “evaluation cultures” (MacKenzie and Spears 2014: 395) are highly analogous.

3 Such as that the risk of being killed by a reactor accident is on the same order of magnitude as being killed by a meteorite (NRC 2004).

4 This is to say that safety in reactors and jetliners is formally expressed as the probability of catastrophic failure, rather than as a function of the consequences of such a failure. The aviation industry’s working definition of safety, for instance, is almost entirely framed in relation to how often crashes will occur, rather than how many people will survive crashes (Lloyd and Tye 1982). The nuclear sphere takes the same approach: since the early 1970s, the NRC’s ‘risk assessments’ have essentially been ‘reliability assessments’ (Rip 1986: 7–9; Fuller 1976: 149–186).

5 When the director of the FAA testified to the Senate that “When we say an airline is safe to fly, it is safe to fly. There is no gray area” (in Langewiesche 1998), he captured an attitude and idiom that pervades the discourse of critical-technology regulation. An attitude that is especially prevalent in nuclear discourse, where the NRC explicitly trains its staff to speak of risk in terms of objective certainty (see, e.g., NRC 2004).

6 For a discussion of how, see Downer (2014).

7 Many of which are articulated and justified more fully in prior research (Downer 2007, 2009, 2011a, b).

8 Such assumptions are not always straightforward. When the US infantry started fighting in Vietnam, for instance, much of the reliability data for its rifles was undermined by changing disciplinary norms and the usually humid operating environment.

9 Engineers can cut this time by running parallel tests, but not even the most extensive test program could plausibly demonstrate the reliabilities demanded of critical systems.

10 Engines, meanwhile, have extra valves, bolts and circuit-boards for the same reason.

11 If one element has a 0.0001 probability of failing over a given time, in other words, then the probability of two redundant elements failing over the same time, is 0.0001^2,^ or 0.00000001: a ten-thousandfold increase (Littlewood et al. 2002: 781).

12 The rules for ‘type-certification’ are encoded in a sprawling network of criteria, stipulations and standards. The minutiae are an extensive pyramid of guidance material—usually Technical Standard Orders—that specify detailed stipulations for each part and system of a civil aircraft, down to the last bolt. At the tip of this pyramid is Part 25 of the code of Federal Aviation Regulations (FAR-25): the master-document governing the design of large civil aircraft as an integrated system.

13 Which is to say that they should function 99.9999999 percent of the time: the oft-cited “nine-9s,” (usually expressed as a probability of 0.999999999, where a value of ‘1’ represents perfection).

14 Birds tend to flock, for instance, so the probability of a bird striking one engine is not ‘independent’ of the probability of a bird striking another.

15 Aeronautical engineers work hard to mitigate such complications—they ‘isolate’ engines by separating them on the wing, for instance—but such efforts are never perfect.

16 Often upwards of 20 hours a day, with periodic breaks for maintenance.

17 Albeit with certain *ceteris paribus* assumptions.

18 A stubbornly suicidal traveler would have to take a random airline flight every day for 19,000 years to stand a better-than-even chance of succumbing to a fatal crash (Barnett and Wang 1998).

19 One need only scan the many ‘jetliner of tomorrow’ concepts that periodically appear in the mainstream press, or simply the innovations that characterize the military sphere, to see that there is nothing inevitable about the standard airframe paradigm. A wide range of technological innovations, such as blended wing-body designs, promise to make air travel cheaper and faster if manufacturers were more daring (or, by the logic of this paper, reckless).

20 If civil aviation designers are the conservatives of the aviation world, then their military counterparts are its radicals. The military’s need to be competitive, combined with its much higher risk tolerance, encourages engineers to accept the uncertainties of innovation in pursuit of marginal combat advantages. In doing so, it builds aircraft that are orders of magnitude less reliable than their civil counterparts, and, almost inadvertently, provides an unofficial test program for civil aviation. The trajectory of military aviation design hints at a path that civil manufacturers might have followed if not constrained by reliability. A jetliner that embraced aeronautical principles found in contemporary military aircraft (such as the blended wing-body design of the B-2 bomber, for example) might offer considerable advantages over its rivals in terms of range, speed, capacity, efficiency and environmental impact (see, e.g., Stewart 2014).

21 Slayton and Spinardi (2016) find that many aviation engineers construe the transition to composites as a radical innovation. For the purposes of this paper, however, it makes little sense to construe innovativeness as an actor’s category, and, if anything, their finding testifies eloquently to the industry’s highly conservative interpretation of innovation.

22 There are, to be sure, uncertainties involved even in the most modest innovation. Subtle differences arising from new configurations or operating environments sometimes prove to be fateful, for example. Yet such uncertainties are much more manageable than those involved in assessing an entirely unproven technology.

23 Boeing, for instance, diligently compiles insights from service experience into a volume—‘*Design Objectives and Criteria’—*which acts as a canonical text in the planning stages of its new jetliners.

24 This is visible, for instance, in the industry’s unusually far-reaching commitment to codifying the lessons of its missteps and false assumptions (e.g., Macrae 2013).

25 This, incidentally, is why airframe manufacturers, who operate in a competitive marketplace, are willing to delay adopting many seemingly advantageous innovations: they recognize that it is essential to reliability and that unreliability, or even the perception thereof, can be a commercial disaster. As Perrow (1999) points out, the aviation industry has structural incentives to favor safety.

26 The demands of ultra-high reliability create a strong ‘path dependency.’

27 The airplane has an uncommon nomenclature, in that it does not receive an article in common English usage (i.e., It is not *The* Concorde).

28 It is telling in this context, that Concorde’s airworthiness certificate was withdrawn immediately after the disaster: grounding the entire fleet for over a year until extensive design modifications had been implemented. A measure that is almost unique in modern civil aviation. It is also worth noting that a contributory factor to the 2000 accident was the fact that the airplane combined multiple engines in single housings: a design decision that regulations expressly forbid in other jetliners.

29 The unhappy history of Concorde’s Russian cousin, the Tupolev Tu-144, only serves to reinforce the point. The airplane entered passenger service in November 1977, and ceased in 1978 after 55 service flights. The airplane accrued two crashes in its lifetime.

30 Both invoke the ‘test-and-multiply’ formula to justify predictive reliability assertions that could not be grounded on tests alone.

31 In theory at least, it should be much more feasible to statistically demonstrate an *insufficient* level of reliability. Major reactor accidents have occurred far more frequently than official reliability assessments have predicted. The exact numbers vary, depending on how one classifies ‘an accident’ (whether Fukushima counts as one meltdown or three, for example), but Ramana (2011) puts the historical rate of serious meltdowns at 1 in every 3,000 reactor years, while Taebi et al. (2012: 203fn) put it at somewhere between one in every 1,300 to 3,600. For an examination of why nuclear safety claims have not been falsified by these events, see Downer (2014).

32 This is the logical basis on which formal reliability studies exclude past accidents from their analyses (Although the same logic should preclude any historical reliability assessment. For it follows that if evidence of accidents is not statistically relevant, then neither is evidence of ‘non-accidents’).

33 Aside from those plants that failed unequivocally, these were indubitably less safe than experts calculated, but the industry enthusiastically contests their relevance.

34 Such as the manner in which metal fatigues between certain rivet configurations, when combined with specific manufacturing imperfections and an unusually moist, saline operating environment (see Downer 2011b).
